# Emerging Regulatory Role of Nrf2 in Iron, Heme, and Hemoglobin Metabolism in Physiology and Disease

**DOI:** 10.3389/fvets.2018.00242

**Published:** 2018-10-10

**Authors:** Shuya Kasai, Junsei Mimura, Taku Ozaki, Ken Itoh

**Affiliations:** ^1^Department of Stress Response Science, Center for Advanced Medical Research, Hirosaki University Graduate School of Medicine, Hirosaki, Japan; ^2^Department of Biological Science, Iwate University, Morioka, Japan

**Keywords:** Nrf2, iron, heme, hemoglobin, oxidative stress

## Abstract

Iron has played an important role in energy production since the beginning of life, as iron-catalyzed redox reactions are required for energy production. Oxygen, a highly efficient electron acceptor with high reduction potential, facilitates highly efficient energy production in eukaryotic cells. However, the increasing atmospheric oxygen concentration produces new threats to the organism, as oxygen reacts with iron and produces reactive oxygen species unless its levels are strictly regulated. As the size of multicellular organisms increases, these organisms must transport oxygen to the peripheral tissues and begin to employ red blood cells containing hemoglobin. This system is potentially a double-edged sword, as hemoglobin autoxidation occurs at a certain speed and releases free iron into the cytoplasm. Nrf2 belongs to the CNC transcription factor family, in which NF-E2p45 is the founding member. NF-E2p45 was first identified as a transcription factor that binds to the erythroid gene regulatory element NF-E2 located in the promoter region of the heme biosynthetic porphobilinogen deaminase gene. Human Nrf2 was also identified as a transcription factor that binds to the regulatory region of the β-globin gene. Despite these original findings, NF-E2p45 and Nrf2 knockout mice exhibit few erythroid phenotypes. Nrf2 regulates the expression of a wide range of antioxidant and detoxification enzymes. In this review article, we describe and discuss the roles of Nrf2 in various iron-mediated bioreactions and its possible coevolution with iron and oxygen.

## Roles of iron and oxygen in energy production

Organisms of all 3 major domains of life (i.e., bacteria, archaea and eukaryotes) produce energy by forming a redox reaction-coupled proton gradient, suggesting an ancestral origin of the energetic mechanism (1). The existence of iron in the evolutionarily conserved cellular respiration systems (i.e., iron sulfur proteins, such as Riske iron-sulfur proteins and heme binding proteins, such as cytochrome C) suggests that iron has had an important role in energy production since the beginning of life (2). As iron is a highly redox-active substance that displays high reactivity with molecular oxygen, the system is susceptible to oxidative stress unless the reactions of iron with oxygen and reactive oxygen species (ROS) are strictly regulated (3). Many antioxidant enzymes and iron storage proteins have evolved to cope with oxidative stress. One of the most important gene regulatory systems that protect against oxidative stress in higher eukaryotes is the Keap1-Nrf2 pathway (4). Here, we review the relationships between the Keap1-Nrf2 system and iron, oxygen.

## CNC family transcription factors and Nrf2

**Figure 1:**
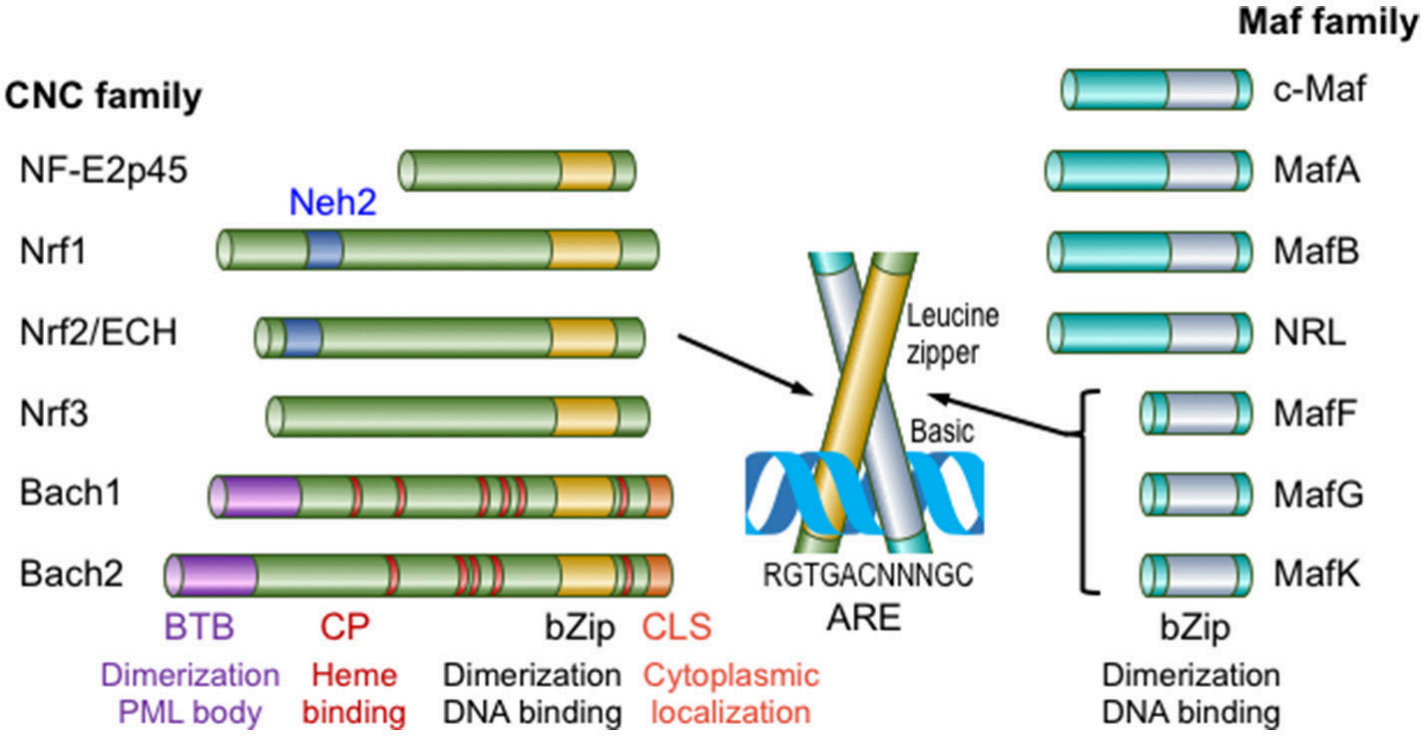
Members of the Maf and CNC transcription factor families in mammals. Maf transcription factors are classified into large Maf proteins, consisting of c-Maf, MafA, MafB, and NRL, and small Maf proteins (sMafs), comprising MafF, MafG, and MafK. The CNC family comprises NF-E2p45, Nrf1-3, Bach1, and Bach2. Only mammalian family members are shown in this figure. CLS, cytoplasmic localization signal. This figure is modified from reference (4).

Nrf2 belongs to the Cap'n'collor (CNC) subfamily of basic leucine zipper type transcription factors; the founding member of which is NF-E2p45 (5) (Figure 1). NF-E2 designates binding activity to the NF-E2 binding site in the gene regulatory regions of various heme biosynthetic genes, such as the porphobilinogen deaminase (*PBGD*) gene, and the enhancer region of the β-globin gene in electrophoretic mobility shift assays (EMSAs). The CNC family includes Nrf1, Nrf2, Nrf3, Bach1 and Bach2 in addition to NF-E2p45. Of these CNC family proteins, Bach1 and Bach2 act as transcriptional repressors and the other proteins act as transcriptional activators. Nrf2 was first identified in humans as a protein that recognizes the NF-E2 binding site of the human β-globin gene (6). We independently cloned chicken Nrf2 (originally named as ECH; *E*rythroid cell–derived protein with *C*NC *H*omology) by screening a peripheral red blood cell (RBC) cDNA library (RBC4) obtained from chickens with phenylhydrazine-induced hemolytic anemia using sequence homology to the mouse NF-E2p45 cDNA (7). Phenylhydrazine is known to induce oxidative stress and cause hemolytic anemia (8). Interestingly, the chicken homolog of NF-E2p45 was not obtained in the screen, but ECH represents a major CNC family protein expressed in peripheral blood RBCs from anemic chickens. The expression of the ECH gene is induced upon the differentiation of chicken erythroid cell line HD3, suggesting that chicken Nrf2 has important roles in maintaining the homeostasis of mature RBCs. Notably, among vertebrates, only mammalian RBCs lost their nuclei during the differentiation into the mature RBCs (i.e., transcription does not occur in mature RBCs). Unexpectedly, mice lacking CNC transcription family proteins exhibit few erythroid phenotypes. Neonatal NF-E2p45 knockout (KO) mice suffer from lethal bleeding due to the lack of platelets caused by abnormal megakaryocyte differentiation (9). Mice who have survived the neonatal lethality exhibit only mild hypochromic anemia with reticulocytosis and splenomegaly. Nrf2 KO mice are fertile and exhibit no signs of anemia (10), although their teeth are whitish brown due to the defect of iron deposition on the tooth surface (11) (discussed later). Furthermore, mice lacking both NF-E2p45 and Nrf2 did not exacerbated phenotype of NF-E2p45 KO mice (12). KO mice of other CNC transcription factors did not show phenotypes related to erythrocyte function (5).

**Figure 2:**
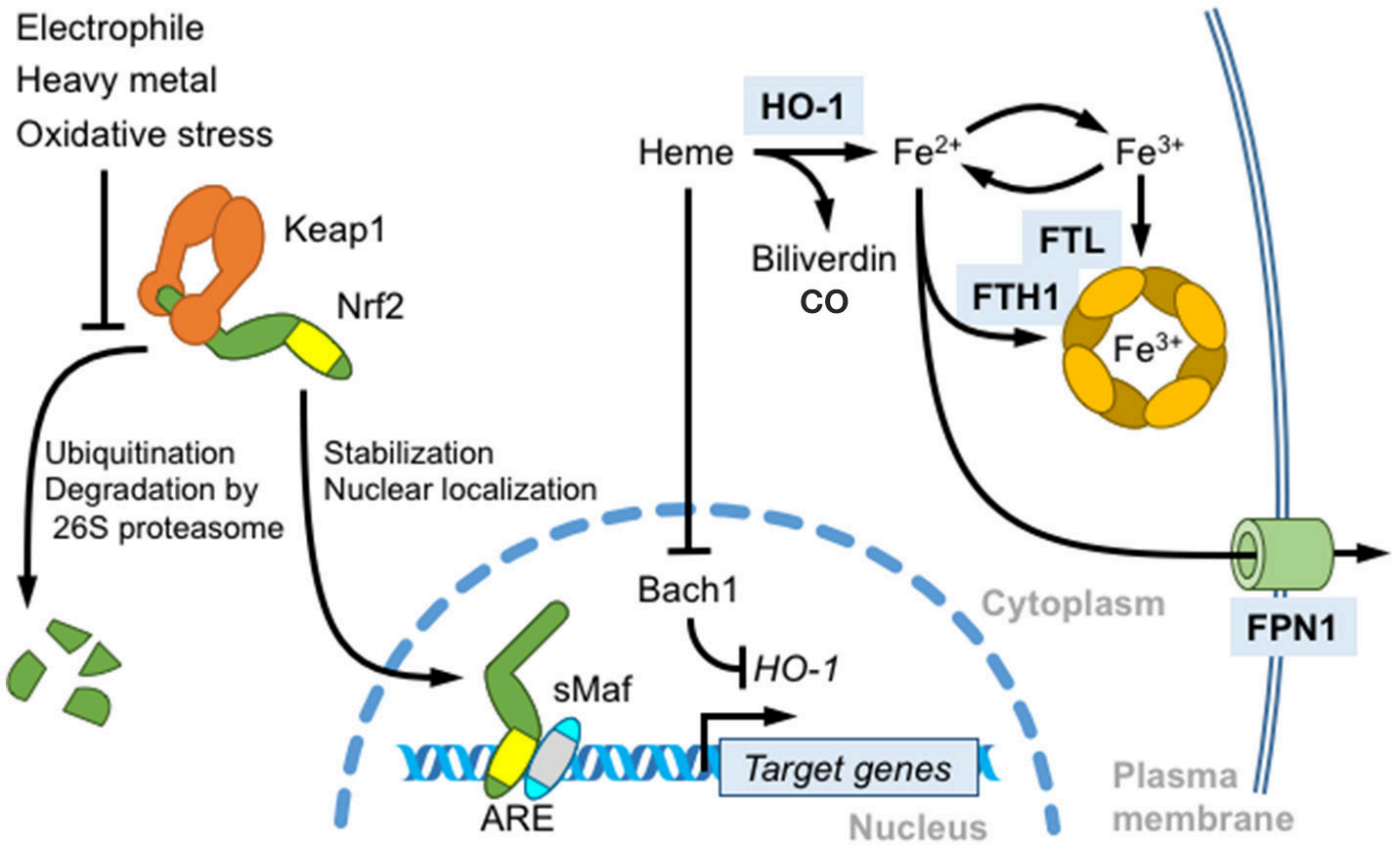
Role of Nrf2 in heme and iron metabolism. Nrf2 is a master regulator of the antioxidant response induced by thiol-reactive substances, such as electrophilic reagents and heavy metals. In unstressed cells, Nrf2 is constitutively ubiquitinated by the Keap1-Cullin 3 ubiquitination complex and degraded by the 26S proteasome. Keap1 oxidation induces Nrf2 stabilization, nuclear translocation, heterodimerization with sMafs, and the transcriptional activation of target genes by binding to antioxidant response elements (AREs). An Nrf2 target gene, HO-1, degrades free heme into biliverdin, carbon monoxide and iron. HO-1 transcription is repressed by Bach1. Heme binding induces the nuclear export and degradation of Bach1. Free iron released from heme also regulates ROS production. Nrf2 induces the transcription of ferritin heavy chain (FTH1) and light chain (FTL) to store iron in the oxidized state and of ferroportin 1 (FPN1) to export intracellular labile iron.

Antioxidant responsive elements (AREs) or electrophile responsive elements (EpREs) are regulatory DNA elements that mediate the gene induction in response to electrophiles and are present in the regulatory regions of genes encoding various phase 2 detoxifying enzymes, such as electrophile-conjugating enzymes (13). Based on the similarity between the NF-E2 binding sequence and AREs, we previously showed that Nrf2 regulates ARE-mediated gene induction in response to electrophiles by generating Nrf2 KO mice (10). Currently, Nrf2 is known to regulate the expression of a wide range of genes related to 1) electrophile detoxification 2) antioxidant synthesis and antioxidant proteins, as well as 3) proteasome subunits, etc. (please refer to the comprehensive reviews (4, 14) for additional details). Nrf2 is mainly regulated by the Keap1- and ubiquitin/proteasome-mediated pathway. Nrf2 is constitutively degraded by the pathway, but upon exposure to electrophiles or oxidative stress, Keap1-mediated Nrf2 degradation is attenuated, leading to Nrf2 stabilization and nuclear accumulation (4, 14) (Figure 2). The extracellular administration of hemin also activates Nrf2 (15). In contrast, Bach1-mediated transcriptional repression is relieved by increasing heme concentration (16). Bach1 negatively regulates the expression of the β-globin and heme oxygenase 1 (*HO-1*) genes, and probably other ARE-containing genes to lesser extent (17, 18). Both Bach1 and Bach2 possess heme-binding CP (Cysteine-Proline) motifs and heme inactivates the Bach1-mediated transcriptional repression. Bach1 nuclear export and degradation are regulated by heme.

## The protective effects of Nrf2 on oxidative stress and infection/inflammation are mediated by iron regulation

Nrf2 regulates a wide range of cytoprotective responses and protects cells against various diseases and toxicities (14). In this review, we will focus on the Nrf2-mediated cytoprotective response achieved by iron regulation and detoxification.

### Overview of systemic iron regulation

Systemic iron homeostasis is maintained by multiple mechanisms (19). Humans contain ~3,500–4,000 mg of iron, and two-thirds of the iron pool is sequestered in the hematopoietic organs such that RBC contains ~2,400–2,500 mg iron (20). Most of the remaining iron is present in the liver (~1,000 mg). Of the iron used for hemoglobin synthesis, 20–25 mg per day of iron is reutilized for RBC production in the spleen. Systemic iron homeostasis is regulated by the liver peptide hormone hepcidin. Hepcidin expression is induced under iron-overloaded conditions. Ferroportin (FPN1, also referred as SLC40A1) is the sole iron exporter in mammalian cells and is expressed at high levels in the basolateral membrane of the small intestine. Hepcidin degrades ferroportin and inhibits iron absorption from the intestine. Macrophages in reticuloendothelial systems scavenge circulating heme and hemoglobin and engulf aged or stressed erythrocytes (erythrophagocytosis) to degrade heme into biliverdin, carbon monoxide and iron via HO-1 activity. Liberated iron is stored with ferritin or exported via FPN1 for utilization in other cells (21) (Figure 2). In particular, HO-1 and FPN1 expressed in splenic macrophages regulate iron reutilization from senescent RBCs for *de novo* erythropoiesis.

### Nrf2 regulates ferritin and ferrroportin1 gene expression

Since free iron readily transforms between Fe^2+^ and Fe^3+^ and is involved in the production of the highly reactive hydroxyl radical (HO^•^) from hydrogen peroxide (H_2_O_2_) (Fenton reaction), the labile iron pool is tightly regulated by multiple mechanisms, including Nrf2-mediated ferritin and ferroportin expression. Excess labile iron is stored in ferritin, which consists of 24 subunits of heavy chain (FTH1) and light chain (FTL) in various ratios and having differing functions. FTH1 possesses ferroxidase activity and stores iron in the stable ferrihydrite form (Figure 2). According to Pietsch et al., Nrf2 activator, β-naphthoflavone and dithiolethione induce FTH1 and FTL expression in wild-type mouse embryonic fibroblasts (MEFs) but not in Nrf2 KO MEFs, and an ARE located 4 kb upstream of the *FTH1* transcriptional start site is responsible for the induction by Nrf2 (22). FTH1 and FTL expression are regulated not only by transcription but also by translation; an iron-responsive element (IRE) located in the 5′-untranslated region of target mRNAs is bound to iron regulatory proteins (IRPs) and negatively regulates its translation in the absence of iron (23, 24). Marro et al. clarified that heme-induced *FPN1* transcription is mediated by Nrf2 activation and Bach1 inactivation in the RAW264.7 murine macrophage cell line (25). The authors identified an ARE motif present 7 kb upstream of the *FPN1* transcription start site that is responsible for Nrf2- and Bach1-mediated *FPN1* gene regulation. Bach1 forms a heterodimer with sMaf and competes with Nrf2 for ARE binding in the absence of heme binding. FPN1 translation is regulated by IRE and IRPs (25), similar to FTH1 and FTL. Furthermore, FPN1 is degraded by the hepcidin-mediated lysosomal pathway. Theurl et al. identified the liver is the primary tissue responsible for disease-associated stressed erythrocyte clearance by transiently differentiated macrophages displaying Nrf2-dependent FPN1 expression (26). The monocytes ingesting stressed erythrocytes are attracted by the chemokines Ccl2 and Ccl3 produced in the liver. Monocytes transiently differentiate into FPN1^+^Tim-4^neg^ macrophages induced by colony stimulating factor 1 (Csf1) produced by Kupffer cells in the liver challenged with stressed erythrocytes. Csf1 induces FPN1 expression in wild-type mice, but not in Nrf2 KO mice, while Csf2 produced in the spleen antagonizes FPN1 induction. Ccr2 and Ccr5 antagonists block the aforementioned response and result in increased labile plasma iron levels and liver injury.

FPN1 is not only important for systematic iron homeostasis, but also involved in certain anti-inflammatory mechanisms. It is previously reported that inflammatory cytokines, such as LPS repress *FPN1* gene transcription in splenic macrophages (27). Furthermore, hepcidin is increased by the inflammatory cytokine, such as IL-6 in the liver and degrades FPN1 in macrophages (28). This FPN1 suppression is proposed to contribute to the anemia of chronic disease by sequestering iron in the macrophages and accumulated iron in the macrophage may accelerates the inflammatory response (29). Furthermore, it is reported that the translation of inflammatory cytokine, such as TNFα and IL-6 in macrophage requires iron (30) and that TRAM/TRIF pathway downstream of TLR4 (31) or TLR4 localization to lipid raft is specifically sensitive to iron depletion (32). Consistently, FPN1 overexpression lowered LPS-induced IL-6 production in J774 macrophages (30). As shown in our previous study, Nrf2 activation induces the expression of FPN1 and another ion transporter, Nramp1, in wild-type bone marrow-derived macrophages (BMDMs), but not in BMDMs from Nrf2 KO mice (33). Although an LPS treatment suppresses *FPN1* transcription in macrophages, Nrf2 activation reversed the *FPN1* suppression in human peripheral blood-derived macrophages and murine peritoneal macrophages (33). Therefore, we proposed that Nrf2 plays an anti-inflammatory role by antagonizing the inflammation-mediated suppression of *FPN1* gene expression. On the other hand, FPN1 is also important for immune response, as iron is required for microbial proliferation. Nairz et al. clarified that Nrf2 is involved in nitric oxide (NO)-induced FPN1 expression and iron efflux to prevent intracellular pathogen proliferation (34). NO is a gaseous radical produced by inducible NO synthase (NOS2 or iNOS) as mediator of the immune response, as well as a toxic effector against infected pathogens. Both in the NOS2^−/−^ spleen and primary peritoneal macrophages derived from NOS2^−/−^ mice, FPN1 expression is decreased and cellular iron content is increased compared to wild-type mice, which are associated with decreased Nrf2 activation. NO donor treatment or *Salmonella enterica* infection induces both Nrf2 activation and FPN1 expression, and the latter is suppressed by a NOS2 inhibitor. NOS2^−/−^ peritoneal macrophages are susceptible to *salmonella* infection, but rescued by the overexpression of Nrf2 or FPN1. Interestingly, *in vivo Salmonella enterica* infection experiment demonstrated that iron chelation rescues the susceptible phenotypes observed in NOS2^−/−^, such as increased bacterial colony formation and reduced inflammatory cytokine production (TNFα, IL-12, and IFNγ) (34).

Sirtuin family proteins belong to the class III histone deacetylases and deacetylate substrate proteins using NAD^+^ as a cofactor. There are 7 sirtuins (Sirts) in mammals and Sirt2 is a cytoplasmic enzyme that regulates various processes including aging, apoptosis and cellular response (35). Yang et al reported that non-heme iron content is decreased in MEFs of Sirt2 KO mice and Sirt2 knockdown HepG2 cells, which is associated with increased *FPN1* gene expression (36). They showed that Sirt2 deacetylates Nrf2 and renders Nrf2 susceptible to the proteasomal degradation. In Sirt2 KO livers, hyperactivated Nrf2 causes FPN1 overexpression and this increase was restored in Sirt2/Nrf2 double KO mice. Sirt2 KO hepatocytes are susceptible to iron deficiency and Sirt2 activity is negatively correlated with iron content in liver biopsies from human fatal neonatal hemochromatosis patients. Thus, the Nrf2-FPN1 axis participates in the cytoprotective effects on iron-mediated oxidative stress and in the host immune response to intracellular pathogens.

### Nrf2-dependent regulation of heme oxygenase 1 and heme toxicity

**Figure 3:**
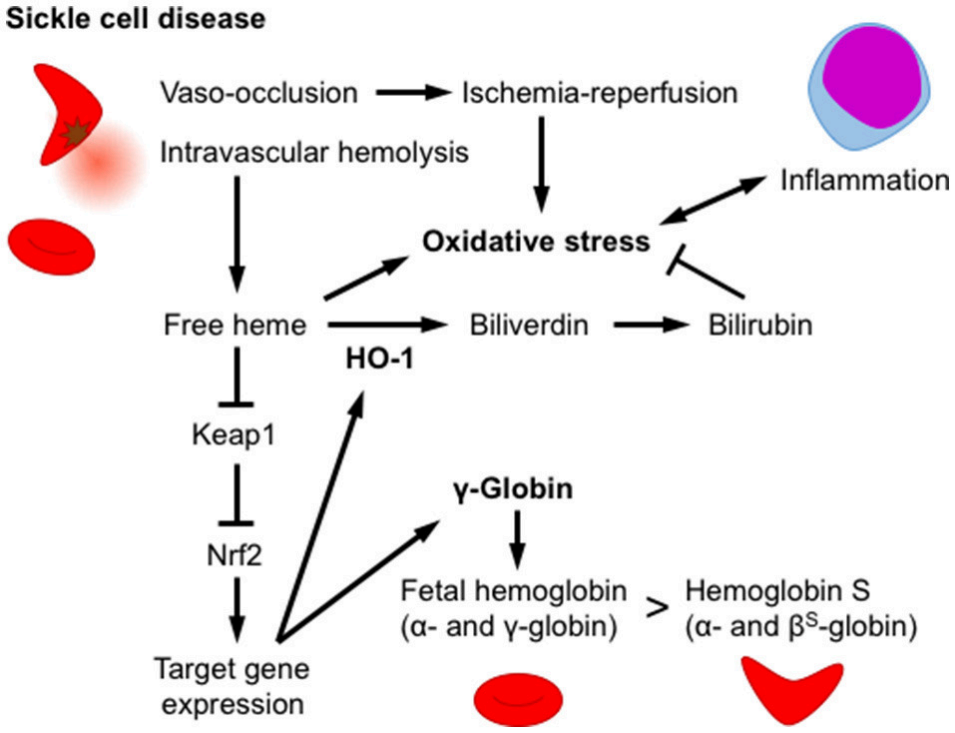
Nrf2/HO-1 counteracts heme toxicity. In sickle cell disease, erythrocytes are deformed by the polymerization of hemoglobin S, and oxidative stress subsequently develops, inducing intravascular hemolysis and vaso-occlusion. An increase in the free heme concentration activates Nrf2 and induces HO-1 expression. HO-1 degrades heme into biliverdin, which is subsequently reduced into the antioxidant bilirubin. Nrf2 activation increases fetal hemoglobin levels by inducing the expression of the γ-globin gene and alleviates hemoglobin S production in erythroid progenitor cells. Nrf2 activation attenuates damage to multiple tissues, as well as the production of inflammatory cytokines.

Heme toxicity is an important pathophysiology manifested by intravascular hemolysis. Sickle cell disease (SCD) is inherited disorder caused by a missense mutation in the β-globin gene (hemoglobin S). A homozygous β-globin gene mutation causes hemoglobin polymerization under hypoxic conditions that deforms red blood cells into a sickle-like morphology (37). Patients with SCD develop chronic intravascular hemolysis, anemia and inflammation, as well as increased vascular occlusion whereas the thalassemia patients, another prevalent hemoglobinopathy, demonstrate intrasplenic destruction of RBCs. It is reported that methemoglobin A or more vigorously hemoglobin S releases free heme (38). Furthermore, it is reported that free heme is increased in sickle cell disease model mouse (39). The free heme released by hemolysis and ischemia-reperfusion injury induces ROS production and subsequently multiple tissue injuries. Townes and colleagues generated mice harboring human α-globin and β-globin genes with or without mutations (β^A^ and β^S^, respectively) as SCD models (40). It is reported that Keap1-flox allele is hypomorphic and mice with this allele (Keap^F/−^) show significant Nrf2 activation and target gene induction, but escape the postnatal lethality of Keap1^−/−^ mice (41). Keleku-Kukwete et al. evaluated the effect of Nrf2 activation on SCD by crossing SCD mice with hypomorphic Keap1 heterozygotes (Keap1^F/−^) (39). Nrf2 activation alleviates liver injury and lung inflammation in SCD mice. Nrf2 activation decreases the serum concentrations of heme, indicating an impact of HO-1 on heme breakdown (Figure 3). In contrast, Nrf2 activation fails to attenuate hemolysis and splenomegaly, a hallmark of stress-induced erythropoiesis in SCD mice (39). The authors also showed that the administration of the Nrf2 activator CDDO-Im improves liver and lung pathology and inflammation in SCD mice, indicating that Nrf2 is a pharmacological target for SCD therapy (39). Hydroxyurea (HU) is currently utilized in SCD therapy to induce the production of γ-globin, a fetal hemoglobin subunit produced during infant liver erythropoiesis, to replace β^S^-globin in adult hemoglobin (42). Although Nrf2 activation by Keap1^F/−^ fails to induce significant γ-globin expression in the spleen, Krishnamoorthy et al. showed that the Nrf2 activator dimethyl fumarate (DMF) induces γ-globin expression in peripheral blood mononuclear cells derived from patients with SCD (43). DMF not only improves the RBC count and morphology but also attenuates inflammatory cytokine production and adhesion molecule expression. Moreover, DMF and HU co-administration induces a further increase in γ-globin expression, and DMF effectively induces fetal hemoglobin production in the cynomolgus monkey (43). Based on these findings, Nrf2 activation exerts beneficial effects by eliminating heme via HO-1 induction and increases the number of erythrocytes containing fetal hemoglobin by inducing γ-globin expression (Figure 3).

### Rodent specific Nrf2 function in iron transport of the enamel organ

The incisors of Nrf2 KO mice are decolorized by defects in iron transport during enamel organ development (11). In the early maturation stage of mouse incisors, FTH1 is expressed in both the papillary cell layer and ameloblasts, and iron accumulates in the ameloblast cytosol. In the late maturation stage, iron accumulation shifts to the plasma membrane on the enamel side and is finally deposited onto the tooth surface, which is characterized by brownish-yellow color. Nrf2 KO mice show decreased levels of the FTH1 protein in the papillary cell layer and reduced iron accumulation in ameloblasts at the early maturation stage. Furthermore, marked degeneration of Nrf2 KO ameloblasts is observed at the late maturation stage, leading to the iron transport defect and the loss of the acid resistance of the teeth. Although the precise mechanisms underlying the tooth phenotype have not been clarified, this finding suggested the susceptibility of Nrf2 KO mice in which iron metabolism is sufficiently proficient to cause oxidative stress.

## Revisiting the roles of NF-E2p45 and Nrf2 in RBCs

### Roles of NF-E2p45 and Nrf2 in red cell homeostasis

**Figure 4:**
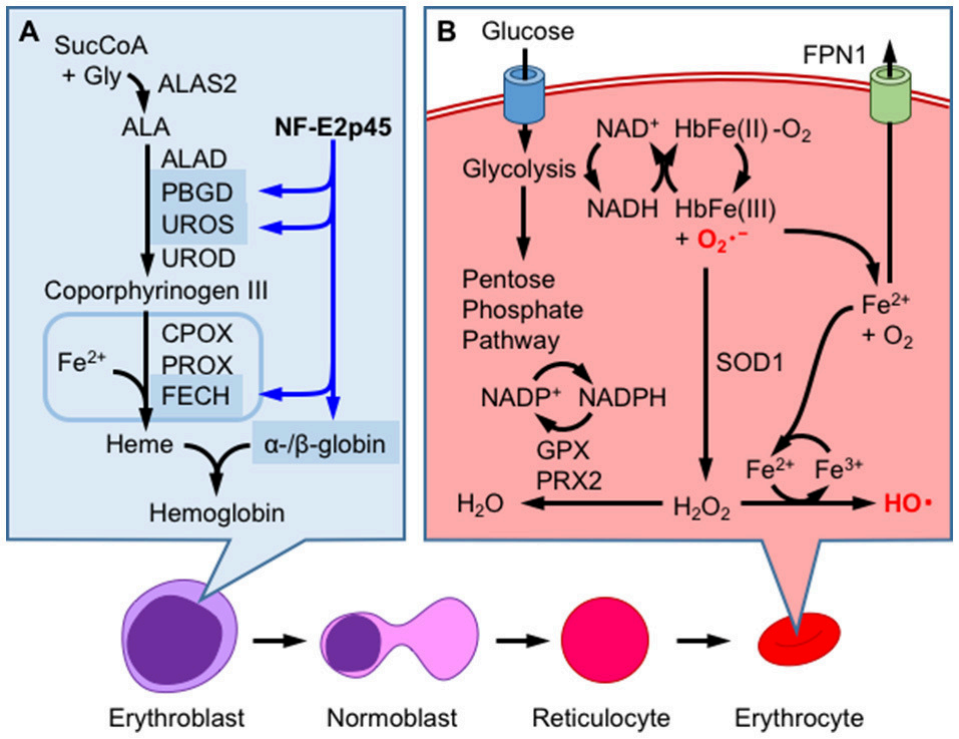
Hemoglobin production and redox homeostasis in erythrocytes. **(A)** NF-E2p45 participates in the transcriptional activation of α- and β-hemoglobin, as well as heme-synthetic enzymes, such as porphobilinogen deaminase (PBGD), uroporphyrinogen III synthase (UROS), and ferrochelatase (FECH), in erythroblasts. After enucleation, erythrocytes combat oxidative stress inducined by hemoglobin autoxidation through a mechanism that is not regulated by gene transcription. **(B)** Glucose metabolism supplies NADH, which is utilized for methemoglobin reduction. Superoxide anions (${\text{O}}_{2}^{•-}$) are eliminated by superoxide dismutase 1 (SOD1), which converts these ions into hydrogen peroxide (H_2_O_2_), or are converted into oxygen through heme degradation. Ferric iron reacts with ${\text{O}}_{2}^{•-}$ by Haber-Weiss reaction to generate highly reactive hydroxyl radical (HO^•^) and oxygen molecule. H_2_O_2_ is further reduced to water by the consumption of NADPH by glutathione peroxidase (GPX) and peroxiredoxin 2 (PRX2); NADPH is produced by the pentose phosphate pathway. FPN1 exports cellular labile iron to prevent ROS production.

**Figure 5:**
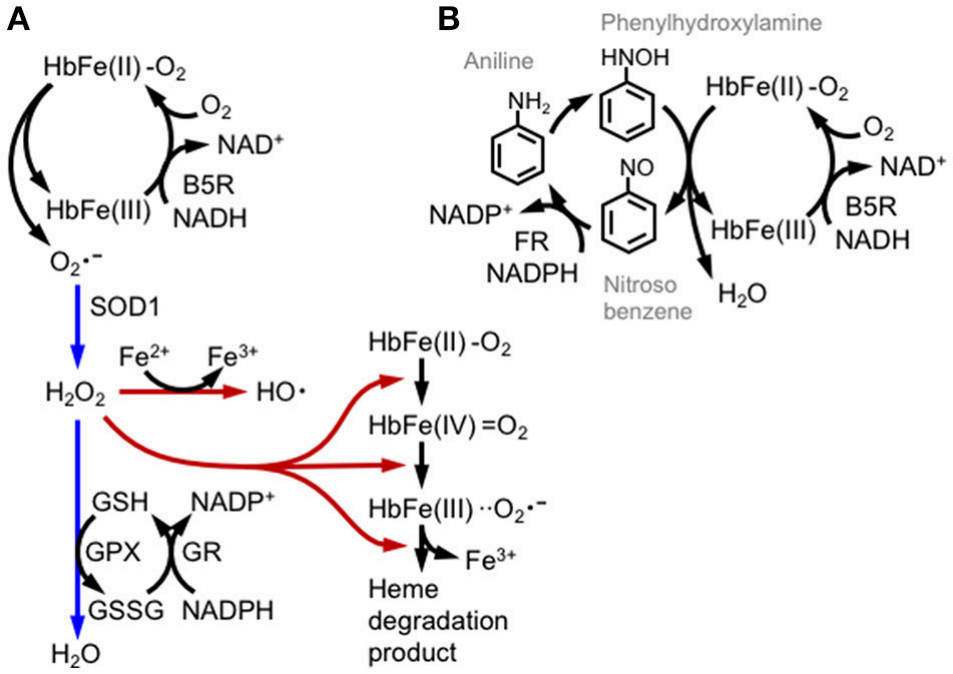
Hemoglobin oxidation and redox catastrophe. **(A)** Oxy-hemoglobin [HbFe(II)-O_2_] is partially converted into methemoglobin [HbFe(III)] and the superoxide anion (${\text{O}}_{2}^{•-}$) by autoxidation. Methemoglobin is then reduced by cytochrome b5 reductase (B5R) using NADH or by glutathione. Superoxide is detoxified by SOD1 and GPX activities (direction illustrated with blue arrows); otherwise hydrogen peroxide (H_2_O_2_) is converted into the hydroxyl radical (HO^•^) by the Fe^2+^ ion or is involved in series of hemoglobin degradation cascades (illustrated with brown arrows). **(B)** Aniline induces methemoglobinemia and hemolysis by repetitive redox cycles of hemoglobin oxidation. Aniline is oxidized by hepatic mixed-function oxidase into bioactive phenylhydroxylamine, which reacts with oxy-hemoglobin and generates nitrosobenzene and methemoglobin. Nitrosobenzene is reduced again by NADPH flavin reductase (FR). Although glycolytic metabolism supplies NADH and NADPH, the phenylhydroxylamine/nitrosobenzene redox cycle produces methemoglobin production and depletes cellular reductants.

Mature RBCs (mRBCs) are particularly susceptible to oxidative stress, as oxygen-bound hemoglobin is oxidized by autoxidation (44, 45) (Figure 4B). During autoxidation, oxygen bound to ferrous-hemoglobin is reduced by iron and generates superoxide. Superoxide is dismutated to hydrogen peroxide through both enzymatic and non-enzymatic processes and reacts with oxygen-bound hemoglobin to generate ferryl-hemoglobin [HbFe(IV) = O_2_, Figure 5A]. The ferryl-hemoglobin subsequently reacts with hydrogen peroxide and is degraded to ferric iron and porphyrin. An estimated 3% of the total oxygenated hemoglobin is oxidized to methemoglobin per day. However, mature RBCs (mRBCs) contain ~1% of methemoglobin, as NADH-dependent cytochrome reductase converts methemoglobin to Fe^2+^-hemoglobin. The liberated ferric iron reacts with superoxide in Haber-Weiss reaction and generates hydroxyl radicals that are toxic to RBCs, mainly due to membrane lipid peroxidation (46) (Figure 4B). mRBCs are equipped with a meticulous network of antioxidant systems to combat these highly oxidative environments (46). Glutathione peroxidase, catalase and membrane localized peroxiredoxin 2 are mainly responsible for detoxifying hydrogen peroxide in RBCs. Furthermore, ferroportin was recently reported to be abundantly expressed in mRBCs and has an important antioxidant function (47). As shown in the study by Zhang et al. of erythroblast-specific FPN1 KO mice, the labile iron pool in erythroblasts is increased, leading to increased hemolysis (47). This observation illustrates the degradation of heme in hemoglobin to ferrous iron in erythroblasts and even in erythrocytes (48).

The oxidation of hemoglobin to methemoglobin is enhanced by various drugs, as observed in subjects with hemolytic anemia caused by a glucose-6-phosphate dehydrogenase (G6PD) deficiency (49, 50). In G6PD-deficient patients, NADPH production is substantially decreased, as mRBCs largely depend on the pentose phosphate pathway for NADPH production. We illustrate how drugs induce hemolytic anemia using the redox active compound aniline (Figure 5B). Aniline is firstly metabolized to phenylhydroxylamine to induce hemoglobin oxidation. Electrons from both ferrous iron and phenylhydroxylamine are transferred to oxygen, generating nitrosobenzene, water and methemoglobin. Nitrosobenzene is reduced back to aniline by flavin reductase using NADPH, or by GSH. Thus, in this reaction, methemoglobin formation is associated with the depletion of GSH and NADPH and therefore generates oxidative stress. Drug-induced hemolytic anemia is prevalent in patients with G6PD deficiencies, and the drugs include the anti-malaria drug primaquine and dapsone (49, 50). Dapsone is diaminodiphenyl sulfone that is metabolized to dapsone hydroxylamine and causes methemoglobin formation and oxidative stress (51). Although RBCs do not regulate transcription, they can determine the basal levels of proteins before enucleation. Indeed, Ter119^+^ RBCs derived from the bone marrow or spleen of NF-E2p45 KO mice accumulate high levels of ROS, and methemoglobin formation is increased after exposure to hydrogen peroxide (52). Methemoglobin formation is enhanced in NF-E2p45 KO mice after the intraperitoneal injection of dapsone, and NF-E2p45 KO mice are more susceptible to phenylhydrazine-induced hemolytic anemia. Furthermore, RBC longevity is reduced in NF-E2p45 KO mice. Interestingly, the expression of GST-Yc and catalase are decreased in the Ter119^+^ RBCs from NF-E2p45 KO mice compared to cells from wild-type mice (52). Selenoproteins are polypeptides that contain selenocysteines and are proficient in redox biochemistry. Importantly, glutathione peroxidase 1 and thioredoxin reductase (which is important for thioredoxin function) are selenoproteins that are mainly responsible for detoxifying ROS in RBCs. In the macrophage- and liver-specific selenocysteine tRNA KO mice, the levels of selenoproteins are decreased as their corresponding mRNAs are degraded by non-sense mediated decay, and conversely Nrf2 is activated to compensate for the oxidative stress in the mice (53). Kawatani et al. extended this observation and showed that Nrf2 and selenoproteins cooperate in the defense mechanism against oxidative stress in RBCs (54). The authors generated compound transgenic mice with a selenocysteine tRNA inducible KO and Nrf2 KO and showed that ROS production was specifically increased in erythroid lineages, but not other hematopoietic lineages, compared to inducible KO mice for the selenocysteine tRNA alone, strongly indicating that Nrf2 regulates RBC redox homeostasis when oxidative stress activates Nrf2, such as in the selenocysteine tRNA KO mice. According to Diederich et al., oxidative stress decreases the deformability of RBCs, and NO ameliorates this defect. Interestingly, the detrimental effect of *tert*-butylhydroperoxide is enhanced in Nrf2 KO RBCs (55). Thus, we surmise that NF-E2p45 regulates the basal expression of antioxidant enzymes and Nrf2 augments the response when Nrf2 is activated in erythroid lineages in response to oxidative stress.

### Roles of Nrf2 and CNC family transcription factors in heme metabolism and globin gene expression—thoughts on evolutionary aspects

Heme-containing proteins, including cytochrome C, hemoglobin, and heme oxygenase, are ancient proteins whose homologs can be identified in bacteria (56, 57). Prototypic hemoglobin may have existed since the evolution of the last eukaryotic ancestor, and several types of hemoglobin or myoglobin-related proteins act as oxygen sensor proteins in bacteria or archaea (58). In multicellular organisms, hemoglobin in RBCs sequesters free oxygen and maintains a low oxygen concentration in tissues. Thus, hemoglobin not only delivers oxygen to peripheral tissues but is also considered a powerful antioxidant, as hemoglobin sequesters oxygen at high blood oxygen pressures (i.e., pVO_2_).

As mentioned above, NF-E2p45 was initially identified as a transcription factor that binds to the NF-E2 site of the *PBGD* gene promoter. Although only a subtle erythroid phenotype of NF-E2 p45 KO mice was initially described (9), the susceptibility of NF-E2p45 KO RBCs to oxidative stress was subsequently reported, as mentioned above (52). Furthermore, abnormal erythroid differentiation is observed in NF-E2p45 KO mice (59). Based on evidence from multiple *in vitro* experiments, NF-E2p45 is an important regulator of the differentiation to the erythroid lineage because it coordinately regulates genes involved in both globin and heme synthesis (see Ref. (60) for a comprehensive review). For example, mouse erythroleukemia (MEL) cells that lack NF-E2p45 (named CB3) do not express globin after the induction of differentiation, but the reintroduction of NF-E2p45 rescues the β-globin deficit (61). Furthermore, the induction of β-globin transcription in MEL cells is associated with NF-E2 biding to the locus control region of the β-globin gene enhancer (62). In addition to globin genes, NF-E2p45 regulates the expression of genes involved in multiple steps of heme biosynthesis in human erythroid cells, particularly the *PBGD, UROD* (63), and ferrochelatase genes (64), the exception is the rate-limiting enzyme ALAS2 (Figure 4A). NF-E2 KO zebrafish exhibit hypochromia in an early developmental stage (i.e., less hemoglobin staining is observed in the embryo) compared to wild-type animals, and a total loss of hemoglobin was observed in response to *tert*-butylhydroperoxide and diquat exposure, while wild-type fish shows only mild hypochromia. The authors argued that hypochromia in NF-E2 KO embryos may be related to decreased ALAS2 expression (48).

Nrf2 also regulates fetal γ-globin gene expression (65) and directly binds to the regulatory region of fetal (HBG1) and embryonic (HBE1) globin (66) genes. Furthermore, Nrf2 binds to the regulatory regions of the various heme biosynthesis genes, including ferrochelatase, although its regulatory role remains to be clarified (66). These results suggest roles for Nrf2 and NF-E2p45 in the expression of the β-globin and heme biosynthetic genes. Although transcriptional regulation does not occur in mature mammalian RBCs, CNC transcription factors may regulate heme and globin synthesis as well as antioxidant gene expression in the erythroblast stages, and contribute to the basal expression observed in mature RBCs (Figure 4B). From the evolutionary perspective, Nrf2 evolution from the ancestral transcription factors occurred around the period of the great oxidation event (67), which was roughly coincident with the period when the metazoan (i.e., predation evolved) and RBCs evolved. Thus, a tempting speculation is that Nrf2 may have evolved from the need for erythrocyte antioxidative function and its role has gradually diminished (i.e., retrogressed) after RBCs lost their nuclei after the branch to mammals. The possibilities remain to be clarified in the future.

## Cross talk between Nrf2 and iron-sulfur cluster formation

Mitochondrial iron-sulfur cluster biogenesis is essential for a number of vital reactions, including respiration, the TCA cycle, and DNA synthesis, among others. Friedreich's ataxia (FRDA) is neuro- and cardio-degenerative disease caused by expansion of GAA repeats in intron 1 of the *FXN* gene and decreased expression of the gene product frataxin. Frataxin is a mitochondrial matrix protein that is indispensable for mitochondrial iron-sulfur cluster synthesis and heme synthesis in concert with iron-sulfur cluster assembly enzyme (ISCU) and ferrochelatase, respectively (68). Decreased frataxin function is thought to decrease iron utilization and cause iron overload in mitochondria. Indeed, iron accumulation has been observed in the brains of patients with FRDA (69), consistent with the observation that a conditional *FXN* knockout (FXN CKO) FRDA mouse model shows mitochondrial iron deposition distinct from the ferritin complex (70). FXN CKO mice exhibit elevated iron contents in tissues and cardiac hypertrophy with downregulation of the genes involved in iron-sulfur cluster biosynthesis (*ISCU*), heme synthesis (*ALAD, UROS*, and *FECH*) and iron transport (*FPN1, FTH1*, and *FTL1*), as well as upregulation of genes involved in iron uptake (*TFR1* and *Sec15l1*) and mitochondrial import (*Mfrn2*) (71–73). An attenuation of Nrf2 activity in the FRDA model has been reported by several groups. The FXN CKO mice show decreased Nrf2 expression in the heart and a concomitant increase in Keap1 expression and GSK3β activation. GSK3β negatively regulates Nrf2 by inducing Nrf2 nuclear export and degradation through the phosphorylation of tyrosine kinase Fyn and Nrf2 itself, respectively (74). Nrf2 activation by oxidative stress is blocked in fibroblasts derived from patients with FRDA (75), as well as in motor neuron-like NSC34 cells expressing an FXN shRNA (76). The defective Nrf2 response in fibroblasts from patients with FRDA is rescued by treatment with Euk134, a cell-permeable catalase mimetic, indicating that chronic H_2_O_2_ generation causes this response (75). Furthermore, in the study by Shan et al., an FRDA mouse model hemizygous for mutant human FXN (YG8R mice) exhibits decreased Nrf2 activity and downregulation of its target genes in dorsal root ganglia (DRG) neurons and in FXN-knockdown cell line models using DRG (ND7/23 cells) and Schwann cells (T265 cells) (77). Interestingly, Sahdeo et al. also observed that dyclonine restores FXN expression by activating Nrf2, and Nrf2 directly binds to the evolutionarily conserved ARE in the upstream region of the *FXN* gene in lymphoblasts from patients with FRDA (78). Although the precise mechanisms of Nrf2 dysfunction have yet to be determined, these results indicate that impairments of Nrf2 function may participate in FRDA pathogenesis by altering the expression of antioxidant and iron metabolism/transport genes.

## Concluding remarks

Since Nrf2 evolved after the branching of metazoans, it has played important roles in the defense mechanisms against oxidative stress and drug detoxification. Nrf2 restricts iron- or heme-mediated oxidative stress by regulating the expression of the FPN1, ferritin and HO-1 genes. Oxidative stress in the erythrocyte may be one of the most important selective forces driving the evolution of members of the CNC transcription factor family, including Nrf2. Currently, Nrf2 activators are being clinically tested in patients with malaria to combat heme-mediated oxidative stress. In the future, we will be able to treat oxidative stress-related diseases by employing Nrf2-mediated iron-detoxification pathways.

## Author contributions

KI designed the structure of the review; SK, JM, TO, and KI wrote the manuscript.

### Conflict of interest statement

The authors declare that the research was conducted in the absence of any commercial or financial relationships that could be construed as a potential conflict of interest.
